# Delirium in the long-term care: a challenge for clinicians and researchers—the negative outcomes related to delirium: a scoping review

**DOI:** 10.1007/s41999-025-01274-0

**Published:** 2025-10-12

**Authors:** Yasushi Takeya, Hidenori Arai

**Affiliations:** 1 Division of Health Science, Graduate School of Medicine, University of Osaka, Osaka, Japan; 2 National Center for Geriatrics and Gerontology, Obu, Aichi Japan

**Keywords:** Delirium, Outcome, Older adults, Long-term care, Nursing home

## Abstract

**Background:**

Most previous studies on the outcomes of delirium have focused on acute care hospital settings. However, the number of residents in long-term care (LTC) facilities is increasing, and these individuals are at risk of experiencing adverse outcomes related to delirium. Recent studies suggest that delirium in LTC settings is associated with serious consequences—such as increased mortality, cognitive decline, and reduced physical function—similar to those reported in acute care. However, high-quality studies specific to this population remain limited.

**Purpose:**

This scoping review aimed to identify adverse outcomes associated with delirium in long-term care settings and to explore appropriate outcome measures for future research.

**Methods:**

This scoping review was conducted in accordance with the Joanna Briggs Institute methodology and adhered to the PRISMA-ScR checklist to identify studies examining adverse outcomes of delirium in LTC settings. MEDLINE, EMBASE, and Web of Science were searched on May 2025.

**Results:**

A total of 14 studies met the inclusion criteria. Delirium was defined and evaluated using a variety of assessment tools, including DSM-IV-based algorithms, the Confusion Assessment Method (CAM), and the Short-CAM (S-CAM). Follow-up durations ranged from 1 week to 2 years. Despite substantial heterogeneity in study designs and outcome measures, multiple studies identified associations between delirium and adverse outcomes, such as increased mortality, cognitive decline, falls, and deterioration in activities of daily living (ADL).

**Conclusions:**

This scoping review provides insight into adverse outcomes of delirium in LTC settings and the inherent heterogeneity across LTC settings.

**Supplementary Information:**

The online version contains supplementary material available at 10.1007/s41999-025-01274-0.

## Introduction

### Outocomes of delirium

Delirium is an acute disturbance in consciousness and cognition frequently observed among older adults. In this population, it has been linked to a variety of serious negative outcomes, including cognitive decline and the onset of dementia [1, 2], reduced physical functioning [3, 4], increased risk of falls [5, 6], pressure ulcers, dehydration, and elevated rates of morbidity and mortality [7–9]. However, most of this evidence is derived from studies conducted in acute care hospital settings. Delirium is also a major concern in LTC facilities, where its prevalence is high and its impact on residents can be substantial. However, research specifically focused on LTC settings remains limited, resulting in an insufficient understanding of delirium within these environments.

### Differences in care settings that influence delirium outcomes

LTC is conceptually distinct from temporary or transitional care services, such as respite care, intermediate care, and post-acute care. These services are designed to provide short-term medical and nursing support during recovery periods or to alleviate caregiver burden. In contrast, LTC refers to long-term or permanent residential care for older adults and individuals with chronic illnesses, in which daily living is closely integrated with ongoing medical and support services. The definitions and terminology related to LTC differ significantly across countries, reflecting variations in healthcare systems, financial structures, and social welfare policies; currently, there is no internationally standardized definition. A key characteristic of LTC research is the pronounced heterogeneity of LTC facilities in terms of service provision, staffing, and resident populations. This heterogeneity directly influences the clinical presentation, management, and outcomes of conditions such as delirium, thereby posing methodological challenges to synthesizing and comparing study findings. Moreover, LTC environments differ fundamentally from acute care hospitals, which focus on rapid diagnosis and life-saving interventions. In acute settings, outcomes such as reduced mortality and short-term functional recovery are often prioritized. In contrast, LTC places greater emphasis on preserving cognitive function, maintaining independence in daily activities, and ensuring residents' comfort and quality of life.

This scoping review aimed to synthesize existing evidence on the adverse outcomes associated with delirium in LTC facilities.

## Methodology

This scoping review was conducted to map the evidence regarding negative outcomes of delirium in LTC settings. The review was developed in accordance with the PRISMA Extension for Scoping Reviews (PRISMA-ScR) Checklist [10]. The PRISMA flow diagram is presented according to Page et al. [11]. No formal protocol was registered for this review.

### Eligibility criteria

The inclusion criteria followed the PCC (population, concept, and context) framework. The Population included older adults receiving long-term care and healthcare professionals involved in LTC (e.g., nurses, care workers, care managers, physicians). The Concept focused on adverse outcomes of delirium, such as falls, death, functional decline, as well as process-related outcomes, including quality of care and healthcare workers’ knowledge or attitudes. The Context covered long-term care facilities, home care, integrated community care, and chronic care hospital wards providing long-term care services. Only interventional studies (RCTs, non-RCTs) and observational studies (cohort, case–control, cross-sectional) were included. Qualitative studies were excluded. Studies that reported only the incidence and prevalence of delirium as outcome measures were excluded from this review.

### Information sources and search strategy

A comprehensive literature search was conducted by a medical librarian at Osaka University on May 5, 2025. Three bibliographic databases—MEDLINE, EMBASE, and Web of Science—were searched. In addition, hand-searching of the reference lists of a related review article was conducted and yielded 3 additional records. No language or publication year restrictions were applied. After removing 419 duplicate records, a total of 2323 records were screened.

The search strategy was developed in collaboration with the librarian and tailored to each database. The complete MEDLINE search string, including Boolean operators, MeSH terms, field tags, and annotation notes, is provided in Supplementary Table S1 to ensure reproducibility. The search was designed to capture literature related to delirium or confusion as exposures, adverse outcomes or process-related outcomes, such as functional decline, death, falls, quality of care, or caregiver burden, and settings consistent with long-term care. The search excluded acute care hospitals and temporary or transitional care services, such as respite care, intermediate care, or post-acute care. Age filters were not applied, but the search focused on populations aged 65 and older through context and setting criteria.

The choice of these three databases is considered appropriate given their comprehensive coverage of medical, gerontological, and health services research. As the review targeted interventional and observational studies reporting adverse outcomes of delirium, MEDLINE, EMBASE, and Web of Science were prioritized over more specialized databases, such as CINAHL or PsycINFO, which are often oriented toward qualitative or psychosocial research. Furthermore, to minimize omissions, reference lists of a related review were hand-searched to identify additional eligible studies.

**Fig. 1:**
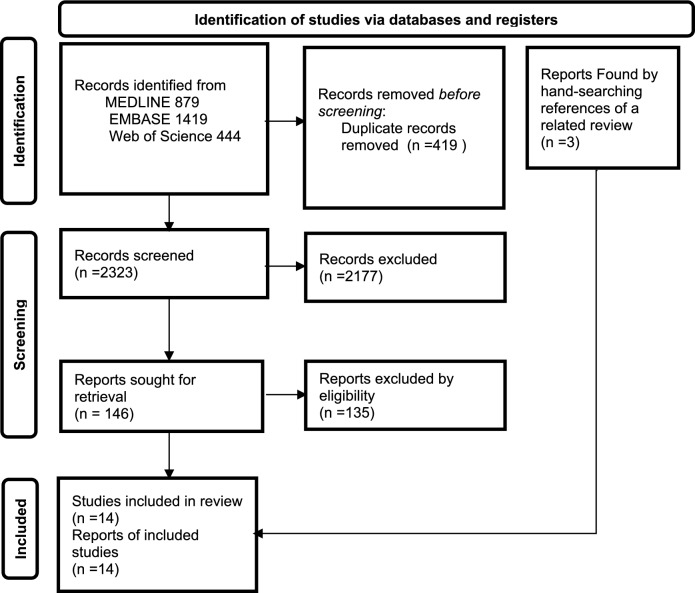
PRISMA flow diagram of study selection

The overall study selection process is illustrated in Fig. 1, a PRISMA 2020-compliant flow diagram adapted from Page et al. [11].

### Selection of sources of evidence

After removing duplicates, two independent reviewers screened titles and abstracts for relevance based on the eligibility criteria. Full texts of potentially relevant articles were retrieved and assessed for final inclusion. Disagreements were resolved through discussion or by consulting a third reviewer.

### Data charting process

A standardized data extraction form was developed and pilot-tested. One reviewer extracted the data, and a second reviewer independently checked the accuracy. Extracted data items included: study objective, study design (observational, RCT, cluster RCT), sample size (exposed/unexposed, control/intervention), intervention or exposure factor, country, study population (age, gender, etc.), setting (e.g., nursing home, hospital), definition of delirium/confusion (assessment tools, diagnostic criteria), follow-up duration, outcome, summary of findings, and assessment scales used (e.g., ADL, quality of life (QOL)).

### Synthesis of results

A descriptive synthesis was performed to summarize the findings. Results were organized according to study design, type of outcome assessed, and setting. Trends, gaps, and areas for future research were highlighted in line with realist principles.

## Results

A total of 14 studies were included in this review: three randomized controlled trials, ten observational studies, and one quasi-experimental study. These studies were conducted in long-term care facilities in the United Kingdom, the United States, Canada, and South Korea. Delirium was defined and assessed using various evaluation tools, including DSM-IV-based algorithms, the Confusion Assessment Method (CAM), Short-CAM (S-CAM), and the modified Nursing Delirium Screening Scale (Nu-DESC-m). Follow-up durations ranged from 1 week to 2 years. Although there was heterogeneity in study designs and outcome measures, many studies reported associations between delirium and adverse long-term outcomes, such as mortality and cognitive decline. Details are provided in Table 1.

**Table 1:** Data extraction table on delirium as described in the included studies

Author (Refs.)	Country	Sample size and characteristics	Study design	Setting	Study objective	Outcome(s)	Follow-up duration	Delirium definition
Murray et al. [12]	USA	N = 325; Mean age 80.5; Hospitalized elderly; From community and LTC	Observational (Prospective cohort study)	Acute care hospital	To determine whether delirium, an acute change in cognitive function, is associated with long-term loss of physical function	ADL change; Functional decline	3 and 6 months	DSM-III criteria via DSI, nurse checklist, and chart review
Lapane et al. [13]	USA	N = 9,223; Mean age 81.5 men; Aged 65 years or older; > 90% Caucasian	Observational (Retrospective cohort study)	Nursing homes	To examine predictors of mortality in a large population-based cohort of men and women with Alzheimer's disease (AD) living in long-term care facilities	Age at death	23 months	Observation: Less alert, awareness change, incoherent speech, lethargy, cognitive fluctuation (daytime)
Kelly et al. [14]	USA	N = 61; Mean age 89; 72% female; All Caucasian	Observational (Prospective cohort study)	Acute-care unit within LTC facility	To assess the severity, course, and persistence of delirium and its relation to in-hospital, 1-month, and 3-month mortality	Mortality (in-hospital, 1 month, 3 months); Delirium persistence	3 months	Assessment: CAM; Clinical observation over 48 h
McCusker et al. [15]	Canada	N = 235; LTC residents aged ≥ 65 years; In 7 facilities	Observational (Secondary analysis of prospective cohort)	LTC facilities	To assess how including nurse-observed delirium symptoms affects prevalence rates and the prediction of outcomes in long-term care settings	Death; Cognitive decline (HDS); Functional decline (Barthel); Composite outcomes	6 months	Assessment: CAM; based on research staff and nurse observations
Decrane et al. [16]	USA	N = 320; Mean age 85.4; 81% female; Rural LTC residents	Observational (Prospective cohort study)	LTC facilities	To investigate the relationship between delirium subtypes and 12-month mortality among elderly LTC residents	Time to death; Cause of death	12 months	Assessment: CAM, MMSE, NEECHAM, CAC-A, Vigilance A; Daily observation by trained nurses
DeCrane et al. [17]	USA	N = 320; Rural LTC residents; Acute illness	Observational (Prospective cohort study)	LTC facilities	To examine falls as an outcome measure at 12 months for two‑group comparison (delirium cases and noncases) and five‑group comparison (noncases, hypoactive, hyperactive, mixed delirium cases, and subsyndromal delirium cases)	h incidence; Time to first fall	12 months	Delirium subtypes + SSD defined via 28-day surveillance; Standardized tools (CAM-based)
Boockvar et al. [18]	USA	N = 136; Mean age 76.2; 60% male; On psychoactive medications	Observational (Prospective cohort study)	LTC facility	To ascertain the incidence of delirium during acute illness in nursing home residents, describe its timing, identify risk factors, and examine its relationship with complications	Delirium incidence; Cognitive and functional decline; Falls	14-day follow-up post illness onset + 14 days post-hospitalization if applicable	Assessment: CAM
Davis et al. [19]	UK	N = 2,197; Median age 77; 64% female; 23% with dementia	Observational (Prospective cohort study)	Community-based setting (including care homes)	To construct an algorithm for the diagnosis of delirium using the Geriatric Mental State (GMS) examination, test its criterion validity against mortality and dementia risk, and report age-specific prevalence	Mortality; Incident dementia (2-year follow-up)	2 years	DSM-IV algorithm with GMS and HAS; requires acute change, fluctuation, and inattention/drowsiness; SSD = ≥ 1 symptom
Siddiqi et al. [20]	UK	N = 215 (IG:CG = 103:112); Mean age 86 (IG), 86 (CG); 69% female; High dementia prevalence	Cluster RCT (Feasibility cluster randomized controlled trial)	Nursing homes	To assess the feasibility of a definitive cluster randomized trial of a delirium prevention intervention in care homes, focusing on recruitment, outcome measurement, and implementation	Delirium occurrence (primary); severity, duration, admissions, falls, mortality, medication use, QoL (secondary)	1 month (delirium); 6 months (hospitalization/death)	Assessment: CAM; DRS-R-98 for severity; supplemented by staff observations and case notes
Moon and Park [21]	South Korea	N = 173; Mean age 76.9; Aged > 18 years; 46% female	Observational (Prospective cohort study)	LTC facility	To investigate the incidence of delirium and delirium superimposed on dementia (DSD) in LTC patients, and examine their associated outcomes	Primary: Delirium incidence, mortality; Secondary: Readmission, discharge placement, length of stay	3 months (delirium); 6 months (mortality)	Assessment: Short CAM (S-CAM); based on CAM criteria
Boockvar et al. [22]	USA	N = 219 (IG:CG = 105:114); LTC residents; 78% female; 52% African–American	Cluster Randomized Controlled Trial (Cluster RCT)	LTC facility	To test the efficacy of the Hospital Elder Life Program adapted for long-term care (HELP–LTC) in preventing delirium	Delirium incidence; CAM-S severity score	6.8 days	Assessment: CAM + CAM-S; Severity scored; Observed daily by blinded research assistants
Jeong and Chang [23]	South Korea	N = 74 practitioners (IG:CG = 51:23); Mean age 55–60/N = 195 patients (IG:CG = 138:57); Mean age 83–88; > 98% female	Quasi-experimental (Pre–post design)	LTC facility	To develop and evaluate a multifaceted and evidence-based delirium educational program for practitioners in nursing home settings, and assess its effects on practitioners' knowledge, confidence, clinical skills, and patient outcomes	Knowledge, confidence (0–100); detection ability; psychiatrist-diagnosed delirium	5 months	Assessment: Modified Korean Nu-DESC-m (5 items + fluctuation); DSM-5 diagnosis by psychiatrist
Park and Moon [24]	South Korea	N = 130 (IG:CG = 65:65); Aged 18 years or older; Majority 65 years or older; ~ 60% female	Randomized Controlled Trial (RCT)	LTC facility	To assess the effectiveness of a web-based application (Web_DeliPREVENT_4LCF) for medical staff to easily access and use a comprehensive delirium prevention management program comprising risk prediction, assessment, and intervention in long-term care facilities with insufficient systems	Delirium incidence; mortality (1 and 3 months)	1 and 3 months	Assessment: S-CAM (Web_DeliPREVENT_4LCF); delirium subtypes; twice daily
Webber et al. [25]	Canada (Ontario)	N = 72,061; Aged 65 years or older; Newly admitted to LTC	Observational (Retrospective cohort study)	LTC facilities	To assess the association between probable delirium and long-term cognitive impairment in long-term care (LTC) residents	Long-term cognitive impairment (after LTC admission)	231 days	Validated CAM-based algorithm mapped to MDS elements; defines probable delirium

### Negative outcomes of delirium in LTC settings

Among the 14 studies included in this review, the most frequently reported outcome was mortality (*n* = 8), followed by cognitive decline (*n* = 4), deterioration in activities of daily living *n* = 4), incidence of falls (*n* = 2), delirium severity (*n* = 3), and composite outcomes (*n* = 1).

Among the eight studies that investigated mortality, five reported an increased mortality rate associated with delirium [14, 19, 21, 24, 25], one reported increased mortality only in male residents [13], and two did not report mortality outcomes in relation to the presence or absence of delirium [15, 16].

Among the five studies that investigated cognitive function, three reported cognitive decline associated with delirium [18, 19, 25], one did not report cognitive outcomes by delirium status [22], and one did not report on cognitive outcomes [20].

Among the five studies that investigated ADL, one reported ADL decline [12], one reported no change [18], two did not report on ADL outcomes [15, 17], and one did not report ADL outcomes by delirium status [21].

Among the three studies that assessed the severity of delirium, two reported no change [22, 24], and one reported worsening severity [14].

Among the three studies that investigated falls, two reported an increase in falls [16, 17], and one reported no change [22].

The one study that assessed a composite outcome (death or decline in HDS or BI) did not report the outcome by delirium status [15].

Although not categorized as negative outcomes, two studies reported process-related outcomes, such as changes in staff knowledge, awareness, and responses [20, 23].

### Variation in assessment tools used in LTC facilities

Among the 12 studies excluding those focusing on process-related outcomes, the most commonly used tool for diagnosing delirium was the Confusion Assessment Method (CAM), which was employed in six studies. Other diagnostic tools included the Short CAM (S-CAM) in two studies, DSM-IV in one study, DSM-III in one study, the Delirium Clinical Assessment Protocol (CAP) in one study, and the Memorial Delirium Assessment Scale (MDAS) in one study; one study did not specify the assessment tool used.

In terms of cognitive function assessment, two studies used the Cognitive Performance Scale (CPS), one study used the onset of dementia as an outcome, one used the Hierarchic Dementia Scale (HDS), and one employed an original assessment method.

For the evaluation of ADL, one study used the Functional Independence Measure (FIM), one used the Minimum Data Set–ADL (MDS–ADL), one used the Barthel Index, one used the modified Katz ADL index, and one used the Nursing Home Activities of Daily Living (ADL) Scale.

Regarding the assessment of delirium severity, one study used the CAM-S (Confusion Assessment Method Severity Score), one used the MDAS, and one did not report the tool used.

## Discussion

### Negative outcomes of delirium in LTC

LTC settings, delirium has been associated with adverse outcomes comparable to those observed in acute care hospitals, including increased mortality, cognitive decline, functional deterioration in ADL, and a higher incidence of falls. However, outcomes that may be more specific to the LTC context—such as hospital transfers—or those particularly valued in these settings—such as QOL—were not identified in the studies included in this review. Similarly, although hospital-based studies have reported that delirium contributes substantially to the emotional burden experienced by patients, caregivers, and healthcare professionals [26–28], analogous evidence from LTC settings was not found. Moreover, while delirium has been linked to elevated healthcare costs in acute care populations [29, 30], no such associations were reported in the LTC literature reviewed.

### Challenges in research on delirium in LTC

Numerous studies have reported the prevalence of delirium in LTC facilities; however, estimates range widely from 1.4% to 70.3%, exhibiting considerably greater variability than those observed in acute care hospital settings [31]. In the same population, reported prevalence has varied between 10.1% and 24.9%, depending on the diagnostic criteria employed (e.g., ICD-10, DSM-II, DSM-III, and DSM-IV) [32]. Such variability may be attributed to differences in diagnostic standards, assessment methodologies, resident health status, and the clinical expertise of assessors [33, 34]. Findings from the present review indicate that the CAM was frequently utilized in LTC settings; however, ensuring diagnostic accuracy requires appropriate training and education. Educational interventions targeting LTC staff have been shown to enhance delirium recognition, improve documentation practices, and foster more proactive attitudes toward medical management [35]. Furthermore, incorporating nurse-led observational assessments of delirium symptoms into diagnostic procedures has been reported to improve detection rates in LTC settings [36]. These process-related outcomes underscore the unique diagnostic challenges faced in LTC environments, where both diagnostic accuracy and access to clinical decision support are more limited compared to acute care hospitals.

Due to substantial heterogeneity in study populations and assessment tools, it was not feasible to synthesize findings through meta-analysis or similar quantitative methods in this review. It is, therefore, important to recognize that the current limitations in conducting meta-analyses in LTC research do not reflect a lack of evidence per se, but rather highlight the methodological complexity inherent to this field.

### Future directions for delirium research in LTC

Against this background, efforts have been made to identify a standardized set of outcomes—known as a Core Outcome Set (COS)—that should be consistently reported in research [37]. A COS specifically developed for studies on the prevention and treatment of delirium among older adults residing in LTC facilities proposes six key outcomes that should be consistently measured and reported [38, 39]. These are: (1) incidence of delirium, (2) delirium-related distress, (3) severity of delirium, (4) cognitive function including memory, (5) hospitalization, and (6) mortality. These outcomes reflect the specific characteristics of LTC settings, which often involve a high proportion of residents with cognitive impairment, and they account for the unique care structures and needs that differ from those in acute care settings. The development and implementation of a COS are expected to promote standardization in delirium research in LTC, enhance the comparability of findings across studies, and improve the accuracy of meta-analyses.

## Limitation

This review employed a clearly defined search strategy to identify quantitative aspects of adverse outcomes of delirium in LTC; however, we acknowledge certain methodological limitations. First, although the search strategy was comprehensive and developed in collaboration with a medical librarian, it was limited to three major databases: MEDLINE, EMBASE, and Web of Science. While these databases cover a broad spectrum of medical and health services literature, other databases such as CINAHL or PsycINFO were not included. However, this limitation is partially mitigated by the fact that our focus was on interventional and observational studies related to adverse outcomes of delirium in long-term care settings—areas that are well-represented in the selected databases. In addition, reference lists of relevant reviews were hand-searched to capture potentially missing studies. Second, no formal quality assessment of the included studies was conducted, in line with the general methodology of scoping reviews. As such, the robustness of individual study findings was not critically appraised. Finally, although efforts were made to include all relevant literature regardless of language, there may have been language or publication bias due to database indexing limitations.

## Conclusion

This scoping review revealed that the evidence regarding adverse outcomes of delirium in LTC settings remains limited, and that the substantial heterogeneity across LTC environments makes it difficult to synthesize results. To generate more robust evidence, further research using common outcome measures is needed. At the same time, in the context of diverse LTC settings, deriving outcomes solely from traditional quantitative studies is challenging, and efforts are underway to develop COS that incorporate insights from qualitative research.

## Supplementary Information

Below is the link to the electronic supplementary material.

Supplementary file1 (DOCX 32 kb)
