# Supplementary material for: Delirium in the long-term care: a challenge for clinicians and researchers—the negative outcomes related to delirium: a scoping review
Source: Eur Geriatr Med. 2025 Oct 12;16(6):1961–70. doi: 10.1007/s41999-025-01274-0 (PMC12743706; doi:10.1007/s41999-025-01274-0)
Supplement: Supplementary file 1 — Supplementary file1 (DOCX 32 kb) [file 41999_2025_1274_MOESM1_ESM.docx]

## Supplementary Table S1. MEDLINE Search Strategy (May 5, 2025)

| MEDLINE (Ovid), 20250505 | |  |
| --- | --- | --- |
| **#** | **Searches** | **Results** |
| 1 | Delirium/ | 13588 |
| 2 | Confusion/ | 5214 |
| 3 | delirium.ti,kw. | 14808 |
| 4 | confusion.kw. or (confusion.ti. and MEDLINE.st.) | 4517 |
| 5 | or/1-3 | 23230 |
| 6 | exp Residential Facilities/ | 60854 |
| 7 | Long-Term Care/ | 29591 |
| 8 | ("long term care" or LTC or LTCs or LTCF or LTCFs or LTF or LTFs or "nursing home*" or "group home*" or "home* for the aged" or "residential" or "skilled nurs* facilit*").ti,kw. | 51814 |
| 9 | or/6-8 | 99456 |
| 10 | 5 and 9 | 486 |
| 11 | *Delirium/ | 11317 |
| 12 | *Confusion/ | 2618 |
| 13 | delirium.ti,kw. | 14808 |
| 14 | confusion.kw. or (confusion.ti. and MEDLINE.st.) | 4517 |
| 15 | or/11-13 | 18897 |
| 16 | exp Hospitals/ | 334461 |
| 17 | exp Hospital Units/ | 147164 |
| 18 | (hospital* or hospice* or inpatient* or patient*).hw,ti,kw. | 3902887 |
| 19 | (postoperative or perioperative or operative or surgical or intensive or icu).hw,ti,kw. | 1550270 |
| 20 | surgery.hw,ti,kw,fs. | 2695510 |
| 21 | or/16-20 | 6601040 |
| 22 | 15 not 21 | 7758 |
| 23 | exp *"Quality of Life"/ | 126787 |
| 24 | *"Activities of Daily Living"/ | 25038 |
| 25 | exp *Hospitalization/ | 108214 |
| 26 | *Health Status/ | 42725 |
| 27 | *Mortality/ | 24200 |
| 28 | *Frailty/et | 242 |
| 29 | *Cognitive Dysfunction/et | 7084 |
| 30 | exp *Dementia/et | 7086 |
| 31 | *Catatonia/et | 367 |
| 32 | *Caregiver Burden/ | 482 |
| 33 | *Comorbidity/ | 4539 |
| 34 | ec.fs. | 455080 |
| 35 | ("core outcome set" or COS).ti,kw. | 2269 |
| 36 | ("quality of life" or QOL or "activities of daily living" or ADL or "daily life activit*" or wellbeing or readmission or admission or mortality or death* or cost or costs or economic).ti,kw. | 749402 |
| 37 | (frail* or decline* or dysfunction* or cognit* or dementia or catatonia or burden*).ti,kw. not MEDLINE.st. | 92861 |
| 38 | or/23-37 | 1381749 |
| 39 | 22 and 38 | 413 |
| 40 | or/10,39 | 882 |
| 41 | remove duplicates from 40 | 879 |
